# Humoral Immune Response of Thai Dogs after Oral Vaccination against Rabies with the SPBN GASGAS Vaccine Strain

**DOI:** 10.3390/vaccines8040573

**Published:** 2020-10-01

**Authors:** Kansuda Leelahapongsathon, Suwicha Kasemsuwan, Tanu Pinyopummintr, Orawan Boodde, Parinya Phawaphutayanchai, Nirut Aiyara, Katharina Bobe, Ad Vos, Virginia Friedrichs, Thomas Müller, Conrad M. Freuling, Karoon Chanachai

**Affiliations:** 1 Faculty of Veterinary Medicine, Kasetsart University, Bangkok 10900, Thailand; fvetkul@ku.ac.th (K.L.); fvetswk@ku.ac.th (S.K.); fvettnp1@yahoo.com (T.P.); fvetorb@ku.ac.th (O.B.); 2 Department of Health, Bangkok Metropolitan Administration, Thapthan 61120, Thailand; parinyavet15@gmail.com (P.P.); nirut2522@gmail.com (N.A.); 3 Ceva Innovation Center, 06861 Dessau–Rosslau, Germany; katharina.bobe@ceva.com (K.B.); ad.vos@ceva.com (A.V.); 4 Institute of Immunology, Friedrich-Loeffler-Institut (FLI), 17493 Greifswald-Insel Riems, Germany; Virginia.Friedrichs@fli.de; 5 Institute of Molecular Virology and Cell Biology, Friedrich-Loeffler-Institut (FLI), WHO Collaborating Centre for Rabies Surveillance and Research, 17493 Greifswald-Insel Riems, Germany; 6 Department of Livestock Development, Bangkok 10400, Thailand; kchanachai@hotmail.com

**Keywords:** dogs, oral vaccination, SPBN GASGAS, neutralizing and binding antibodies, immunoglobuline isotypes

## Abstract

Applied research is crucial in pushing the boundaries and finding a solution to the age-old problem of dog-mediated rabies. Although oral vaccination of dogs is considered to have great potential in mass dog vaccination campaigns and could have far-reaching benefits, it is perhaps the most ignored of all available tools in efforts to eliminate dog-mediated rabies, not least because of limited data on immunogenicity, efficacy, and safety of potential oral rabies vaccine candidates. In this study, the long-term immunogenicity in local Thai dogs after oral administration of the highly attenuated 3rd generation rabies virus vaccine strain SPBN GASGAS was assessed. The oral rabies vaccine was administered to dogs by either direct oral administration (*n* = 10) or by offering a vaccine loaded intestine bait (*n* = 15). The humoral immune response was then compared to three groups of dogs; a group that received a parenteral delivered inactivated rabies vaccine (*n* = 10), a group offered a placebo intestine bait (*n* = 7), and a control group (*n* = 4) for an observation period of 365 days. There was no significant difference in the immune response of dogs that received oral and parenteral vaccine in terms of magnitude, kinetics, and persistence of both rabies virus (RABV) neutralizing (RFFIT) and binding (ELISA) antibodies. Although the single parenteral injection of an inactivated rabies vaccine mounted a slightly higher humoral immune response than the orally delivered live vaccine, RABV specific antibodies of both types were still detectable after one year in most animals for all treatment groups and resulted in no difference in seropositivity. Characterization of rabies specific antibodies revealed two main classes of antibodies involved in the immune response of dogs vaccinated. While IgM antibodies were the first to appear, the succeeding IgG response was mainly IgG2 dominated independent of the vaccine type used. The results support the view that SPBN GASGAS induces a sustained detectable immune response in local dogs both after direct oral administration and via bait application.

## 1. Introduction

Rabies has been threatening humans for millennia, and despite progress in disease prevention and control, tens of thousands of humans still die of rabies, mostly infected by rabid dogs [1]. A global initiative has set the plan to eliminate dog-mediated human rabies by 2030 [2,3]. While human rabies prevention is the priority, the most cost-effective approach—in a true one health concept—is the elimination of the disease from its animal source [4]. Mass dog vaccination (MDV) using inactivated vaccines and delivered via the parenteral route has been the primary approach to rabies control in dogs. However, outside of Latin America where this approach has been used successfully [5], sustained success of MDV in Africa and Asia is scarce. Besides the need for additional resources to achieve the elimination of dog-mediated rabies by 2030, technological progress is also essential [6]. To this end, the concept of oral rabies vaccination (ORV) of dogs has gained renewed interest [7,8,9,10,11]. The added value of ORV of dogs as a complementary tool to mass parenteral dog vaccination campaigns has both quantitative and qualitative considerations; not only does it increase the overall vaccination coverage in the dog population, it specifically targets the free-roaming dog population [7,12,13]. In many areas, the majority of free-roaming dogs cannot be restrained and vaccinated by the parenteral route without special effort and are, therefore, considered inaccessible for rabies vaccination. Thus ORV can compensate for the inadequacies of the parenteral vaccination methods to reach facets of the dog population that are critical to the enzootic transmission of dog-mediated rabies [14]. Hence, field and laboratory studies focused on ORV in dogs have been initiated once more.

Recent fieldwork on ORV has focused on bait development and acceptance [15,16,17,18]. Additionally, due to the fact that all commercially available oral rabies vaccines are based on live replication-competent viruses, an assessment of human safety aspects associated with ORV of dogs was conducted, with particular focus on potential human contacts with the vaccine construct candidates [19]. Finally, the economic aspects of including ORV of dogs has been assessed in detail under specific settings [13,20,21].

In contrast, data on immunogenicity and efficacy of oral rabies vaccines in the target free-roaming dog population are limited and older. In some of these publications, a clear difference in immune response was observed between laboratory dogs and free-roaming dogs indigenous to endemic settings. It was observed that indigenous dogs required much higher doses to develop detectable rabies virus (RABV) neutralizing antibodies (rVNA) [22]. Additionally, sometimes dogs vaccinated by the oral route did not develop detectable levels of rVNA but subsequently survived a rabies challenge infection [23,24,25,26,27].

Due to the paucity of data on the immunogenicity of currently available 3rd generation oral rabies vaccines in local target populations of free-roaming dogs, a long-term immunogenicity study (1-year post-vaccination) in local Thai dogs using the oral rabies vaccine construct SPBN GASGAS was carried out. SPBN GASGAS is a derivate of SAD L16 (cDNA clone of the oral rabies virus vaccine strain SAD B19), which lacks the pseudogene (Ψ) and shows alterations in the gene encoding for the RABV glycoprotein at both amino acid (aa) positions 194 and 333 where all three nucleotides were changed; position 194—AAT [Asn] → TCC [Ser], position 333— AGA [Arg] → GAG [Glu] [28]. The insertion of an additional identical glycoprotein containing the same genetic modifications resulted in a reduced potential risk of reversion to virulence and enhancement of apoptosis [29].

While the immunogenicity and efficacy of this construct have been intensively studied in foxes (*Vulpes vulpes*) and raccoon dogs (*Nyctereutes procyonoides*) [30,31], in the study reported here, we focused on the humoral immune response in domestic dogs. We aimed to gain more detailed insights into (i) the development and kinetics of rabies specific antibodies by determining both rVNA and rabies binding antibodies (rVBA), and (ii) the measured seropositivity achieved in oral treatment groups depending on the assay used. Finally, a subset of the samples was investigated for dynamics of antibody formation, e.g., immunoglobulin isotypes. These results obtained from orally vaccinated dogs using a genetically engineered replication-competent RABV vaccine (SPBN GASGAS) were compared with those of dogs that were vaccinated via the parenteral route using a commercially available inactivated rabies vaccine, dogs given placebo baits, as well as unvaccinated control animals. Although the measurable rabies specific antibody response in orally vaccinated dogs was only slightly lower than in parenterally vaccinated animals, the response in terms of persistence and the temporal course was similar. Both vaccines induced an IgG2-response predominantly—IgG1 levels were low for both vaccine types.

## 2. Materials and Methods

### 2.1. Animals and Housing

**Figure 1:**
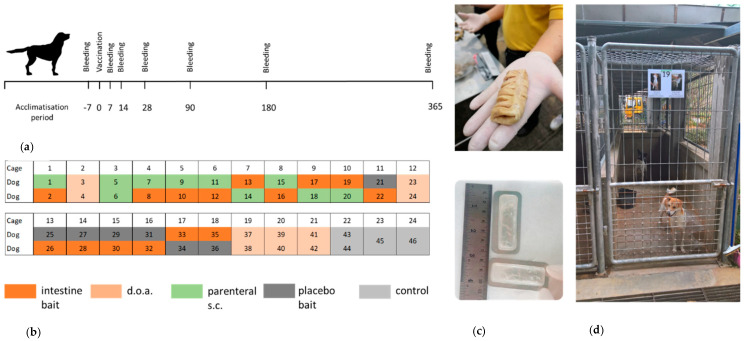
Study design and animal husbandry. (**a**) Outline of the in vivo experiments with an observation period of 365 days. (**b**) Cage allocation of individual animals according to treatment groups: (Group A—blue [bait], Group B—purple [doa], Group C—orange [sc], Group D—red [placebo] and Group E—green [control]). (**c**) Locally produced boiled pig intestine bait (upper picture) and vaccine sachets inserted in a previously cleaned and boiled section of the cow intestine (lower picture). (**d**) Standard animal cages at the dog shelter in Taptan, Uthai-Thani. Cages had a size of 90 × 350 × 190 cm (length × width × height) accessible through an open fenced door. The floor and lower part of the walls (100 cm) was made from concrete, and the upper part of the walls consisted of fencing.

A total of 46 young dogs (26 males and 20 females) were kept at the study site, Bangkok Metropolitan Administration’s dog shelter in Taptan, Uthai-Thani province, Thailand. Thus, the dogs had never received rabies vaccination before the study. The study was approved by Kasetsart University’s Institutional Animal Care and Use Committee (ACKU 61-VET-011). Animals were vaccinated with a combination vaccine (RECOMBITEK^®^ C8, Merial (Thailand) Ltd., Bangkok, Thailand) against canine distemper, parvovirus infection, adenovirus infection, bronchitis, and leptospirosis when puppies were 2, 3, and 4 months of age. They also received helminthic treatment when they were around 3-months-old. The enclosure consisted of 20 individual roofed cages meeting appropriate spacing standards (Figure 1d). The animals were fed once per day with commercial dog food (Blue’s canine special, BLUEFALO Co. Ltd., Nakhon Pathom, Thailand), and water was offered ad libitum. Cages were cleaned every day.

### 2.2. Study Design

The outline of the experiments with an observation period of 365 days is depicted in Figure 1a. The dogs were random allocated into 5 groups: Group A—dogs receiving the oral vaccine inserted in pig intestine bait (bait, *n* = 15), Group B—dogs receiving the oral vaccine by direct oral administration (doa, *n* = 10), Group C—dogs vaccinated by the parenteral route (sc, *n* = 10), Group D—dogs receiving a placebo bait and kept in the same cage as vaccinated dogs (placebo, *n* = 7), and Group E—non-vaccinated naïve control dogs (control, *n* = 4). Two dogs were caged together, except for 2 control dogs that were kept individually (Figure 1b). Dogs were aged 3–12 months (average 7 months) at the starting point of the study (Table S1).

The dogs were uniquely identified by photos placed on the door of each cage and by microchips (Ornthana Intertrade Co., Ltd., Pichit, Thailand) implanted 7 days before the rabies vaccination.

### 2.3. Vaccination

For oral vaccination, the oral rabies vaccine strain SPBN GASGAS was used. The vaccine virus with a titer of 10^8.2^ FFU/mL, filled in sachets (3.0 mL), transported to Thailand and stored frozen (<−20 °C) till the day of vaccination. Each vaccine sachet was inserted in a previously cleaned and boiled section of pig intestine (Figure 1c) before being offered to an individual dog (bait group). For the doa group, 3.0 mL of vaccine virus was slowly released into the oral cavity of the dog using a needleless syringe (Supplementary material). For dogs vaccinated by the parenteral route (sc), a commercial local-available product (Bayovac*R, Bayer Thai Co. Ltd., Bangkok Thailand) was used.

### 2.4. Sampling and Testing Procedure

Dogs were tested for the presence of antibodies against rabies 7 days before vaccination to ensure their naïve status for rabies antibody. Subsequently, blood samples were collected from all animals on 7, 14, 28, 90, 180, and 365 days post-vaccination (dpv) (Figure 1a). Dogs were manually restrained and muzzled while blood was collected from the cephalic vein. Blood samples were centrifuged within 24 h of collection and stored at −20 °C until testing. The level of rVNA and rVBA was determined for all samples taken. For the more detailed analysis of isotypes, blood samples from 6 animals that received a vaccine bait (Group A) and from 6 animals vaccinated by the parenteral route (Group C) were selected randomly. Furthermore, all blood samples from the 4 control animals (Group E) were included.

### 2.5. Diagnostic Assays

The presence of rVNA was determined by using a modified Rapid Fluorescent Focus Inhibition Test (RFFIT) using the challenge virus standard (CVS-11) as a test virus with titers expressed in IU/mL, as described previously [32]. Blood samples were also tested for rVBA using a commercial blocking ELISA (O.K. Servis BioPro, Prague, Czech Republic) according to the manufacturers’ instructions [33], whereby the level of antibodies was expressed in the percentage of inhibition. This percentage was calculated by using the absorption value of the test sample and of a negative control measured at 450 nm. For the ELISA and RFFIT, the cut-off for seropositivity was set at ≥40% inhibition and to ≥0.5 IU/mL, respectively. The seroconversion rate was used to indicate the proportion of samples that met the specified cut-offs.

Rabies virus-specific immunoglobulin classes were comparatively investigated in sera of selected dogs using a cell-based ELISA. Briefly, BHK-21 (baby hamster kidney) derived BSR-T7/5 cells were cultivated in 96 well plates (seeding density per well: 4 × 10^4^ cells) and infected with the vaccine strain SPBN GASGAS used for oral and parenteral vaccination at a multiplicity of infection (MOI) of 1. After 48 h, infected cells were fixed with 4% formaldehyde for 30 min and subsequently incubated with 5% skimmed milk in PBS-T (1xPBS + 0.05% Tween-20) to block nonspecific binding sites. After washing, cells were incubated with diluted sera (1:100 in PBS-T) for 1 h at room temperature (RT) to allow antigen-specific Ig binding. After an additional washing step, dog-specific, horseradish-peroxidase (HRP) labeled Ig antibodies (goat-α-dog-IgA, goat-α-dog-IgM, goat-α-dog-IgG, goat-α-dog-IgG1, goat-α-dog-IgG2, Bethyl Laboratories INC; Montgomery, USA), were added and incubated for 1 h at RT. Antibodies were used at 1:10,000 in PBS-T, according to the manufacturer’s instructions. After washing, Tetramethylbenzidine (TMB) substrate (Merck, Darmstadt, Germany) was added and incubated at RT for 15 min in the dark. Optical density (OD) was measured at 450 nm using a conventional ELISA-Reader.

### 2.6. Statistical Analysis

Differences in mean percent blocking (MPB) values and geometric mean titers (GMT) of VNAs between treatment groups at different sampling time points were tested for significance using unpaired T-tests with a significance level of α = 0.05. Differences in seroconversion rate between different treatment groups at different time points were tested using Chi^2^- or Fisher’s exact test. Sex and age between the different treatment groups were tested using Chi^2^ (sex) and a one-way ANOVA with unequal sample size (age), while the effect of sampling time point and route of administration was tested using a two-way ANOVA test. Differences with *p* < 0.05 were defined as significant. All statistical analyses were carried out using Graphpad Prism 7 (GraphPad Software Inc., San Diego, CA, USA).

## 3. Results

### 3.1. Bait Acceptance and Safety of the Vaccine

Although the dogs were not familiar with the intestine baits, all dogs accepted the baits. Most of the dogs ate the entire intestine bait. A few animals chewed on the bait for a long period (>3 min) and left the remains on the floor of the cage. Three dogs did not discard the perforated sachet due to it being swallowed. No vaccine-induced adverse reactions were observed in any of the dogs during the entire observation period post-vaccination, nor were any health complications seen in the three dogs that swallowed the perforated sachet. One dog (No. 34) in the placebo group (Group D) that died post-treatment was attributed to parasitic anemia.

### 3.2. Rabies Specific Immune Response

**Figure 2:**
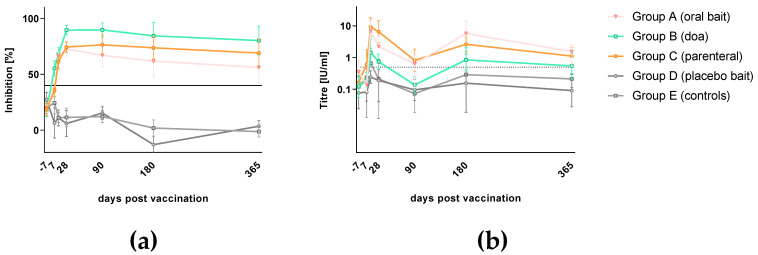
Antibody response in local Thai dogs after oral rabies vaccination (Group A and B), parenteral vaccination (Group C) using SPBN GASGAS and a commercial inactivated vaccine, respectively, as well as after placebo baiting (Group D) and left untreated (Group E). Serology results of all treatment groups are presented as mean percent inhibition and geometric mean titer with standard deviation as measured by ELISA (**a**) and Rapid Fluorescent Focus Inhibition Test (RFFIT) (**b**), respectively.

All dogs tested negative for the presence of rabies antibodies (ELISA) prior to treatment (−7 dpv), while two dogs tested positive in RFFIT (Tables S2 and S3). Oral vaccination with SPBN GASGAS both by doa and via bait induced a strong rabies specific primary immune response as measured both by ELISA and RFFIT (Figure 2) at comparable levels with parenteral vaccination with Bayovac*R. A difference between parenteral as opposed to oral vaccination was only seen in the temporal dynamic: Parenteral vaccination induced a more rapidly detectable antibody response than vaccination by the oral route, which was statistically significant (*p* = 0.0003 [oral] and *p* = 0.004 [sc]). All dogs vaccinated by the parenteral route (Group C) already had detectable rVBAs 7 dpv, as compared to 32% of the orally vaccinated dogs. The great majority of animals (96%) in the orally vaccinated groups (Group A, B) turned positive in ELISA only after 14 dpv (Table S2 and Table 1, Figure 2a). The mean percent inhibition (MPI) was highest in the parentally vaccinated group (Group C), followed by dogs that received a vaccine bait (Group A) and those vaccinated doa (group B) and only slightly declined until the end of the study (365 dpv).

The development of rVNAs followed almost the same pattern but was delayed by approximately 7 days. While all parenterally vaccinated dogs (Group C) showed rVNAs greater than 0.5 IU/mL 14 dpv, 72% of the orally vaccinated dogs (Group A, B) had developed rVNAs above this level at this time point. On average, dogs vaccinated parenterally (Group C) had higher geometric mean titers (GMTs) than the orally treated groups (Group A, B), while among the latter, dogs receiving the vaccine by offering a bait (Group A) had higher GMTs than dogs receiving the same vaccine by doa (Group B). The route of administration (bait, doa, or sc) did not have a significant effect on rVNA-level (*p* = 0.38). The GMTs in all vaccinated groups declined from 14 dpv onwards but were still above the threshold of positivity (0.5 IU/mL) after 365 days (Table S3 and Table 1, Figure 2b). However, there was heterogeneity in the immune response and decline of rVBAs and rVNAs in individual dogs in the different treatment groups. The dogs offered a bait had a much wider variation in rVNA levels than the dogs offered the vaccine doa (Tables S2 and S3). Control dogs (Group E) and the majority of dogs receiving a placebo bait (Group D) did not develop a rabies specific immune response throughout the observation period. Individual values above the threshold for positivity in both ELISA and RFFIT were interpreted as unspecific reactions of serum components.

### 3.3. Kinetics of Immunoglobulin Classes and Isotypes

**Figure 3:**
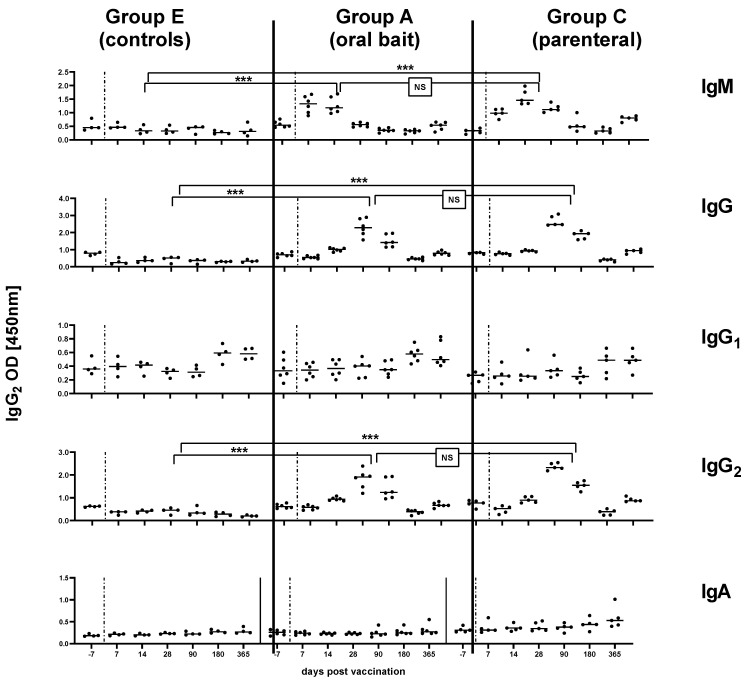
Kinetics of RABV specific immunoglobulin classes (IgM, IgG, IgA) and IgG isotypes (IgG1, IgG2) in the serum of naïve dogs (Group E) compared to orally (Group A) and parenterally vaccinated dogs (Group C) at different time points (days) pv. Each dot represents one individual animal with the median (solid line) depicted for each time point and each group. The vertical dashed lines represent the time point of vaccination. Statistical comparisons of the means for IgM (14 dpv), IgG (28 dpv), IgG_2_ (28 dpv) between the different groups are indicated (Fisher’s t-test, *p* < 0.05, *** = extremely significant p: 0.0001 to 0.001; NS: not significant).

**Figure 4:**
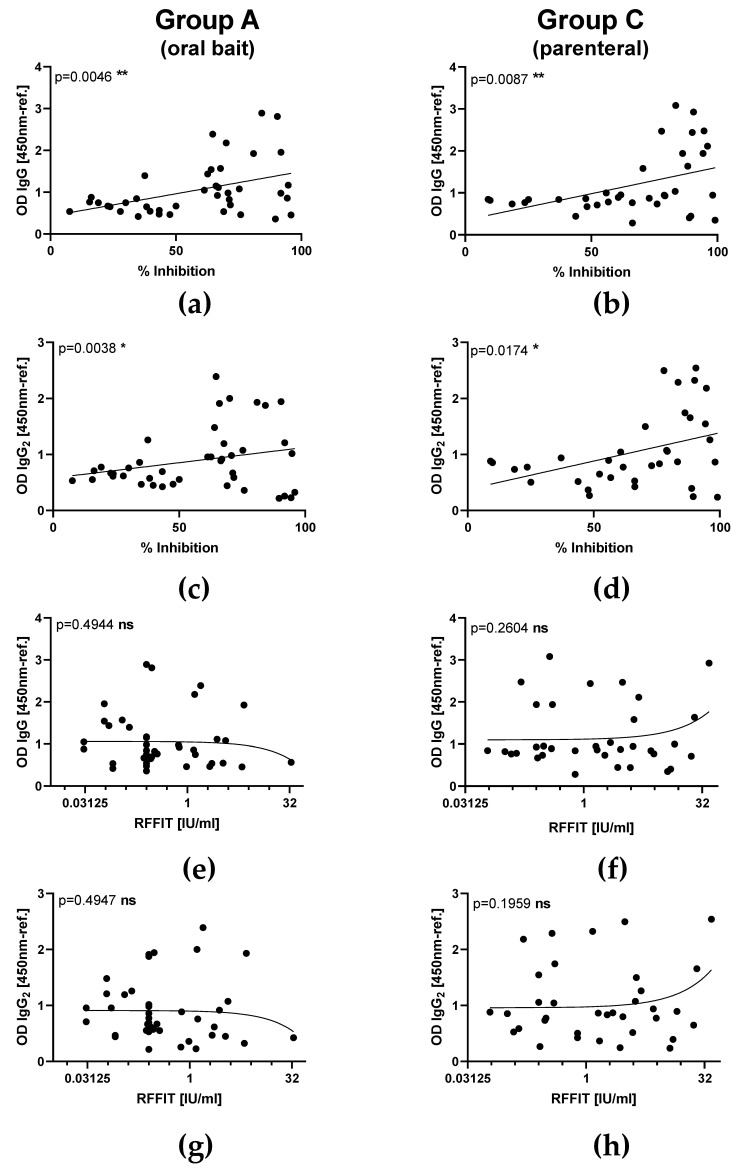
Comparison of correlation between optical density (OD) values of RABV specific immunoglobulins (IgG isotypes) and binding as well as VNAs after parenteral vaccination with an oral rabies vaccine (SPBN GASGAS) between orally (Group A and B) and parenterally (Group C) vaccinated animals. Correlation of RABV-specific IgG (**a**,**b**), and IgG2 (**c**,**d**) with rVBA as measured by ELISA. Correlation of RABV-specific IgG (**e**,**f**), and IgG2 (**g**,**h**) with rVNAs as measured by RFFIT. Each dot represents the results of one individual animal at different time points pv. Linear regression lines (solid lines) and the *p*-values are indicated.

In subset of sera used to immunoglobulin classes and isotypes, in both orally and parenterally vaccinated animals, RABV-specific IgM antibodies were measured as early as 7 dpv and peaked at 14 dpv before decreasing, while IgG antibodies were first above threshold 14 dpv and peaked by 28 dpv. The latter immunoglobulin class was predominantly of the IgG2 subtype independent of the vaccine type. There was no indication for IgG1 or IgA specific immune responses in serum samples from all groups (Figure 3). The OD level for IgG and IgG2 and the corresponding percentage inhibition of rVBA as obtained in ELISA correlated significantly in both the orally and parenterally vaccinated (Group C) animals (Figure 4a–d), while there was no correlation of isotypes with rVNAs (Figure 4e–h).

### 3.4. rVBA Versus rVNA

**Figure 5:**
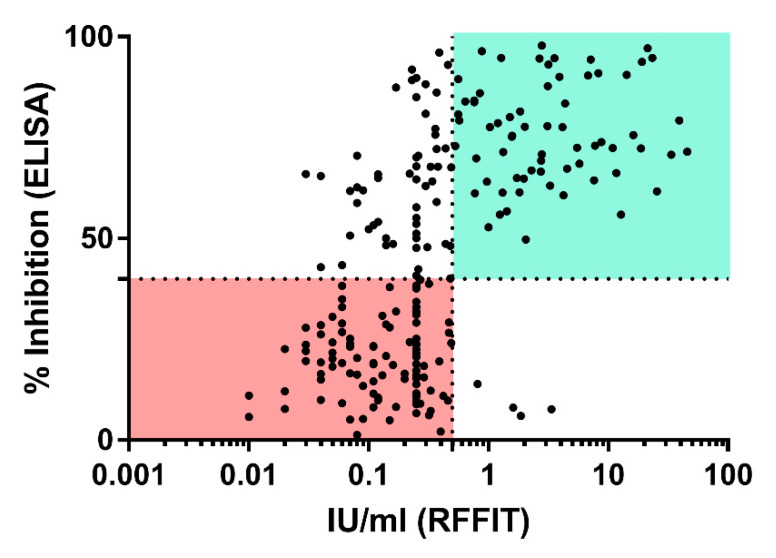
Correlation between ELISA (y-axis) and RFFIT (*x*-axis) results. The dotted lines represent the threshold of positivity in ELISA (horizontal; 40% PI) and RFFIT (vertical, 0.5 IU/mL). Sectors from both assays that are considered negative and positive are highlighted in light green and light red, respectively.

Comparison of the results between the two assays revealed a high number of samples that were ELISA positive (rVBA) but RFFIT (rVNA) negative, while four sera were RFFIT positive but ELISA negative (Figure 5). In the latter group, two animals tested positive prior to vaccination (Group A, C). Furthermore, one dog from the placebo and one from the control group (Group D, E) were RFFIT positive at one sampling time point, while all preceding and subsequent samples taken from these 2 dogs tested negative in RFFIT. Hence, these four samples should be regarded as false positives.

### 3.5. Seroconversion Rates

Comparison of the seroconversion rates within each treatment group as determined by the two serological assays at different sampling points pv (Table 1) revealed no significant difference (*p* > 0.05) between animals vaccinated orally by offering a bait (Group A) or receiving the vaccine by doa (Group B, Table S4).

When the oral vaccination groups were combined, the seroconversion rates were compared to the parenterally vaccinated group (Group C, Table S3). While seropositivity, as obtained by RFFIT at a threshold of >0.5 IU/mL, was significantly higher in the parenterally vaccinated dogs (Group C) at most time points. No difference in seroconversion rate between parenterally and orally vaccinated dogs (Group A, B) could be observed at any time point pv except 7 dpv using the ELISA. Of note, at the end of the observation period at 365 dpv, the majority of orally immunized dogs were still ELISA positive, and no statistical difference of the seropositivity compared to group C was seen, neither for ELISA nor for RFFIT (Table S3).

## 4. Discussion

Our study provides the first insights into the long-term immunogenicity (365 dpv) of the 3rd generation oral rabies vaccine construct SPBN GASGAS in local target dog populations in Thailand after a single vaccine dose application. The results demonstrated that SPBN GASGAS induces a sustained detectable antibody response in local dogs both after doa and via bait application. In a similar study with dogs in Thailand, a vaccine bait containing a vaccinia recombinant vaccine expressing the RABV glycoprotein (V-RG) was offered to dogs [34]. However, the immune response was less pronounced than with the vaccine virus used in our study. In the former study the GMT of the VNA-level one-year post vaccination was only 0.25 IU/mL, and only 58% of the dogs tested >0.1 IU/mL.

Using SPBN GASGAS, the seroconversion after oral vaccination was not significantly different from parenterally vaccinated animals using the ELISA. The fact that orally vaccinated animals had lower titres is most likely a result of the limited total vaccine uptake of the highly attenuated oral rabies vaccine virus SPBN GASGAS by the oral route. This is likely to diminish the immunogenic potential of the vaccine due to a relatively low level of virus replication and spread at the entry site before it is cleared by the immune response [35,36].

Since two different routes of administration (oral vs. parenteral) and two different types of vaccine (live-attenuated vs. inactivated) were used (Groups A, B, C), which may evoke different immunogenic mechanisms [37], we were interested to further characterize the immune response in terms of immunoglobulin classes and IgG subtypes. Comprehensive serological analyses were also applied using ELISA and RFFIT as described previously using the same vaccine construct [30,31]. In our study, we could not detect any differences between parenteral versus oral vaccination groups; in both, the immune response was characterized by a primary phase of IgM followed by IgG antibody production. An IgG1-response was not evident following vaccination with either live oral or inactivated RABV, however, both vaccines induced a strong IgG2 signal (Figure 3). Additionally, total IgG and IgG2 levels at the sampling times corresponded well with peaks of rVNA/rVBA (Figure 2). The fluctuation of GMTs seen in RFFIT post-vaccination as opposed to the stable trend in ELISA is likely due to the biological variance of the RFFIT rather than a true effect of immune stimulation.

Different thresholds for positivity in the RFFIT for various species have been defined [32]. For dogs, it was shown that rVNA levels greater than 0.1 IU/mL were associated with protection [38]. In our study, we defined a level of 0.5 IU/mL as the threshold for positivity in RFFIT, which is recognized by regulatory authorities from most rabies-free areas as proof of adequate immune response to vaccination [39]. In addition, setting the cut-off level at 0.5 IU/mL increases specificity and reduces false-positive results. Nevertheless, there is still the possibility of nonspecific inhibitors to be presented in the sera [40], as exemplified by serum samples of animals from the placebo and control group (Group D, E; Table S2).

Our study confirms previous experimental findings that the rVBA-response is more reliable in demonstrating a successful vaccination attempt than rVNA [32]. Similar results were also obtained during a field study with SPBN GASGAS in Haiti [41].

When using a replication-competent virus vaccine, it is of utmost importance to find the delicate balance between safety and efficacy. None of the dogs vaccinated with SPBN GASGAS showed any adverse reactions during the entire 1-year observation period. Additional safety studies in laboratory dogs (dissemination, overdose) further underscored the high level of attenuation attained with this vaccine strain [42]. The absence of seroconversion in placebo-treated naïve animals, which were co-housed with orally vaccinated dogs (Figure 1), supports evidence that the risk of horizontal transmission of SPBN GASGAS is negligible [42].

The primary objective of oral vaccination is to increase the vaccination coverage among dogs that are inaccessible to parenteral vaccination or only accessible with considerable efforts [7,15]. Taking this into account, the added value of oral vaccination would still be apparent even if the immunogenicity and efficacy were slightly below that of parenteral rabies vaccination. SPBN GASGAS appears to be a promising oral vaccine candidate for dogs and has demonstrated an almost equal immunogenicity to the parenteral vaccine. At the end of the study, the antibody level in several vaccinated animals dropped below the cut-off for seropositivity. This decrease in antibodies is most likely a result of a shift from the production of circulating antibodies to memory B-cells and long-lived plasma cells in the bone marrow that will be activated upon re-exposure to the virus antigen [43,44]. Based on previous vaccination studies that included serological monitoring and a challenge infection [32], it is highly likely that SPBN GASGAS confers immunity and protection for at least one year after oral vaccination in dogs. In this context, and by adhering to the 3R concept of animal welfare it is debatable whether there is a need to demonstrate the protectivity of the elicited immune response in dogs through a challenge infection.

## 5. Conclusions

Our study demonstrates that SPBN GASGAS offered orally to local Thai dogs elicits an immune response that is comparable to parenterally vaccinated dogs. These results support the application of ORV with SPBN GASGAS vaccine strain to complement mass dog vaccination campaigns in the global effort to control and eventually eliminate dog-mediated rabies.

## Figures and Tables

**Table 1:** Seroconversion rates post-vaccination based on both ELISA and RFFIT results.

		Groups				
dpv	Assay	A SPBN GASGAS (bait)	B SPBN GASGAS (doa)	C Bayovac * R (sc)	D Placebo Bait	E Control
−7	ELISA	0/15	0/10	0/10	0/7	0/4
	RFFIT	1/15	0/10	1/10	0/7	0/4
7	ELISA	5/15	3/10	10/10	1/7 *	0/4
	RFFIT	0/15	0/10	4/10	0/7	0/4
14	ELISA	14/15	10/10	10/10	0/7	0/4
	RFFIT	10/15	8/10	10/10	0/7	1/4
28	ELISA	15/15	10/10	10/10	0/7	0/4
	RFFIT	8/15	7/10	10/10	1/7	0/4
90	ELISA	14/15 **	10/10	10/10	0/6	0/4
	RFFIT	4/15	1/10	5/10	0/6	0/4
180	ELISA	14/15	9/10	10/10	0/6	0/4
	RFFIT	8/15	6/10	10/10	1/6 ***	0/4
365	ELISA	13/15	8/10	9/10	0/6	0/4
	RFFIT	7/15	4/10	7/10	0/6	0/4

* Value of positive sample was just above cut-off; 42.9% inhibition, ** Value of negative sample was just below cut-off; 37.6%, *** Extreme high value of positive sample, 6.8 IU/mL, d.p.v.: Days post vaccination; d.o.a = direct oral application, sc = subcutaneously.

## Supplementary Materials

The following are available online at https://www.mdpi.com/2076-393X/8/4/573/s1, Table S1: Sex and age allocation (months) among the four treatment groups. Table S2: ELISA results (percent inhibition) of individual animals in the respective treatment groups at different sampling points post-vaccination. Table S3: RFFIT results (IU/mL) of individual animals in the respective treatment groups at different sampling points post-vaccination. Table S4: Statistical comparison of differences in seroconversion ratios at different time points.

## Abbreviations

mdv	mass dog vaccination
ORV	oral rabies vaccination
dpv	days post vaccination
doa	direct oral application
sc	subcutaneously
rVNA	Rabies virus neutralizing antibodies
rVBA	Rabies virus binding antibodies
RFFIT	Rapid Fluorescent Focus Inhibition Test
ELISA	Enzyme-linked Immunosorbent Assay
