# Supplementary material for: Humoral Immune Response of Thai Dogs after Oral Vaccination against Rabies with the SPBN GASGAS Vaccine Strain
Source: Vaccines (Basel). 2020 Oct 1;8(4):573. doi: 10.3390/vaccines8040573 (PMC7711832; doi:10.3390/vaccines8040573)
Supplement: Supplementary file 1 [file vaccines-08-00573-s001.zip › Table S1.docx]

**Table SX:** Sex – and age allocation (months) among the 4 treatment groups. There was no significant sex (Chi² = 7.352, p=0.12) and age (one-way ANOVA with unequal sample size – p=0.73) differences between the different treatment groups

|  |  | **Group A**  **(bait)** | **Group B**  **(d.o.a.)** | **Group C**  **parenteral (s.c.)** | **Group D**  **placebo** | **Group E**  **control** |
| --- | --- | --- | --- | --- | --- | --- |
| sex | male/female | 10/5 | 2/8 | 7/3 | 4/3 | 3/1 |
| age  (months) | average range | 7.2 3.0 - 12.4 | 5.8 3.2 – 12.4 | 6.7 3.3 – 12.4 | 7.4 4.6 – 12.4 | 6.6 3.6 – 8.7 |
