# Supplementary material for: Humoral Immune Response of Thai Dogs after Oral Vaccination against Rabies with the SPBN GASGAS Vaccine Strain
Source: Vaccines (Basel). 2020 Oct 1;8(4):573. doi: 10.3390/vaccines8040573 (PMC7711832; doi:10.3390/vaccines8040573)
Supplement: Supplementary file 1 [file vaccines-08-00573-s001.zip › Table S2.docx]

Table S1: ELISA results (percent inhibition) of individual animals in the respective treatment groups at different sampling point post vaccination. Positive sera are highlighted in red bold.

|  |  | **ELISA % inhibition** | | | | | | |
| --- | --- | --- | --- | --- | --- | --- | --- | --- |
|  |  | **days post vaccination** | | | | | | |
| **Treatment group** | **Animal ID** | **-7** | **7** | **14** | **28** | **90** | **180** | **365** |
| **A  SPBN GASGAS bait** | 2 | 1.32 | 34.95 | **61.39** | **77.64** | **89.49** | **87.58** | **91.19** |
|  | 8 | 9.59 | 31.87 | **48.64** | **67.80** | **85.06** | **77.89** | **68.81** |
|  | 10 | 15.57 | **47.61** | **70.76** | **90.41** | **94.76** | **95.84** | **94.37** |
|  | 12 | 19.54 | 26.79 | **51.21** | **67.58** | **62.97** | **56.40** | **52.63** |
|  | 13 | 7.73 | 28.48 | **55.08** | **70.49** | **65.86** | **72.05** | **72.96** |
|  | 16 | 16.40 | **53.26** | **71.48** | **75.66** | **81.51** | **81.28** | **73.51** |
|  | 17 | 7.62 | 23.77 | **61.31** | **64.04** | 37.58 | **43.37** | 29.97 |
|  | 19 | 30.79 | **42.35** | **73.88** | **80.11** | **89.78** | **89.54** | **90.64** |
|  | 22 | 38.32 | 39.72 | 34.28 | **67.77** | **62.66** | 34.98 | 22.84 |
|  | 26 | 18.73 | 31.87 | **72.31** | **87.72** | **94.64** | **91.95** | **90.30** |
|  | 28 | 28.95 | 20.36 | **61.20** | **78.56** | **77.19** | **79.12** | **70.67** |
|  | 30 | 23.18 | 30.58 | **49.73** | **67.89** | **64.97** | **60.72** | **42.95** |
|  | 32 | 19.08 | 27.86 | **66.91** | **64.63** | **80.91** | **75.81** | **71.27** |
|  | 33 | 23.85 | **43.32** | **66.53** | **70.05** | **65.95** | **69.05** | **71.67** |
|  | 35 | 16.24 | **50.01** | **75.24** | **84.21** | **91.85** | **89.63** | **91.78** |
|  | mean | 18.46 | 35.52 | 61.33 | 74.30 | 76.35 | 73.68 | 69.04 |
| **B  SPBN GASGAS d.o.a.** | 3 | 9.01 | 29.18 | **67.25** | **77.71** | **54.06** | **52.04** | **43.71** |
|  | 4 | 13.84 | 38.74 | **63.07** | **83.92** | **80.72** | **77.80** | **65.66** |
|  | 23 | 20.59 | **50.07** | **71.41** | **75.69** | **48.63** | **40.19** | 34.13 |
|  | 24 | 21.47 | 37.88 | **61.92** | **66.03** | **50.72** | **57.33** | **50.25** |
|  | 37 | 26.20 | 20.19 | **57.68** | **64.01** | **61.78** | **48.89** | **60.98** |
|  | 38 | 19.59 | 32.71 | **69.88** | **86.05** | **89.23** | **84.70** | **84.42** |
|  | 39 | 24.05 | 28.67 | **55.87** | **59.01** | **65.48** | 26.72 | 21.28 |
|  | 40 | 19.23 | **48.14** | **72.49** | **72.88** | **87.42** | **78.06** | **79.18** |
|  | 41 | 22.51 | **40.81** | **69.28** | **64.80** | **58.78** | **72.12** | **64.92** |
|  | 42 | 22.06 | 38.25 | **68.49** | **75.49** | **72.12** | **79.19** | **56.70** |
|  | mean | 19.85 | 36.46 | 65.73 | 72.56 | 66.89 | 61.70 | 56.12 |
| **C  Bayovac** | 1 | 9.11 | **66.25** | **79.22** | **94.72** | **94.31** | **99.06** | **98.13** |
|  | 5 | 11.62 | **70.84** | **72.30** | **93.74** | **96.45** | **88.40** | **69.41** |
|  | 6 | 13.88 | **52.78** | **64.36** | **97.17** | **94.63** | **93.24** | **90.50** |
|  | 7 | 12.07 | **40.07** | **64.97** | **83.76** | **79.24** | **83.81** | **77.10** |
|  | 9 | 9.84 | **56.73** | **61.67** | **90.52** | **88.26** | **88.87** | **78.92** |
|  | 11 | 18.89 | **53.61** | **72.37** | **90.95** | **97.79** | **96.50** | **96.53** |
|  | 14 | 23.65 | **52.31** | **55.89** | **77.88** | **70.47** | **43.83** | 37.07 |
|  | 15 | 18.61 | **48.28** | **60.68** | **83.46** | **96.09** | **89.47** | **83.24** |
|  | 18 | 32.93 | **64.13** | **77.61** | **93.07** | **93.03** | **94.38** | **94.60** |
|  | 20 | 25.10 | **47.80** | **72.98** | **90.04** | **86.20** | **66.29** | **76.07** |
|  | mean | 17.57 | 55.28 | 68.20 | 89.53 | 89.65 | 84.38 | 80.16 |
| **D  Placebo bait** | 21 | 29.05 | **42.86** | 18.33 | 26.62 | 18.18 | 14.39 | -1.95 |
|  | 25 | 11.00 | 25.12 | 13.37 | 5.74 | 5.08 | 4.67 | -8.42 |
|  | 27 | 20.84 | 23.66 | 15.34 | 21.63 | 9.92 | -2.26 | 5.53 |
|  | 29 | 23.14 | 23.29 | 5.27 | 8.19 | 15.16 | -1.89 | -3.85 |
|  | 31 | 22.57 | 31.04 | 8.93 | 8.08 | 16.07 | 1.36 | 0.86 |
|  | 34 | 16.01 | 11.49 | 9.81 | 7.23 |  |  |  |
|  | 36 | 17.22 | 13.38 | 6.62 | 2.14 | 8.13 | -5.00 | -0.29 |
|  | mean | 19.98 | 24.41 | 11.10 | 11.37 | 12.09 | 1.88 | -1.35 |
| **E  Control** | 43 | 24.21 | 14.53 | 12.28 | -4.36 | 16.52 | -7.63 | 7.04 |
|  | 44 | 27.97 | 10.92 | 16.48 | 10.79 | 10.55 | -4.12 | 0.17 |
|  | 45 | 32.93 | 4.88 | 8.91 | 11.16 | 15.00 | -22.82 | 5.24 |
|  | 46 | 24.28 | -4.87 | 6.02 | 6.16 | 19.08 | -17.43 | 1.49 |
|  | mean | 27.35 | 6.36 | 10.92 | 5.94 | 15.29 | -13.00 | 3.49 |
