# Supplementary material for: Humoral Immune Response of Thai Dogs after Oral Vaccination against Rabies with the SPBN GASGAS Vaccine Strain
Source: Vaccines (Basel). 2020 Oct 1;8(4):573. doi: 10.3390/vaccines8040573 (PMC7711832; doi:10.3390/vaccines8040573)
Supplement: Supplementary file 1 [file vaccines-08-00573-s001.zip › Table S3.docx]

Table S2: RFFIT results (IU/ml) of individual animals in the respective treatment groups at different sampling point post vaccination. Positive sera are highlighted in red bold.

|  |  | **RFFIT virus neutralizing antibody titre in IU/ml** | | | | | | |
| --- | --- | --- | --- | --- | --- | --- | --- | --- |
|  |  | **days post vaccination** | | | | | | |
| **Group** | **Animal ID** | **-7** | **7** | **14** | **28** | **90** | **180** | **365** |
| **A  intestine bait** | 2 | 0.08 | 0.06 | **1.82** | **1.03** | **0.56** | **5.63** | **3.17** |
|  | 8 | 0.25 | 0.25 | 0.44 | 0.38 | 0.25 | 1.65 | 0.25 |
|  | 10 | 0.29 | 0.25 | **33.58** | **6.81** | **1.28** | **3.65** | **2.14** |
|  | 12 | 0.39 | 0.06 | 0.25 | 0.49 | 0.30 | **0.90** | 0.41 |
|  | 13 | 0.02 | 0.04 | 0.25 | 0.08 | 0.12 | 0.40 | 0.09 |
|  | 16 | 0.04 | 0.11 | **45.70** | **16.25** | **1.84** | **5.42** | **1.93** |
|  | 17 | **3.36** | 0.25 | **1.31** | **0.97** | 0.25 | 0.25 | 0.25 |
|  | 19 | 0.13 | 0.26 | **8.75** | **1.51** | 0.25 | 0.25 | 2.32 |
|  | 22 | 0.25 | 0.27 | 0.25 | 0.33 | 0.08 | 0.06 | 0.30 |
|  | 26 | 0.11 | 0.17 | 0.44 | **3.14** | **3.56** | **4.84** | **3.90** |
|  | 28 | 0.06 | 0.08 | **0.77** | **1.20** | 0.36 | **61.82** | 0.40 |
|  | 30 | 0.11 | 0.05 | **2.05** | 0.25 | 0.12 | 0.25 | 0.10 |
|  | 32 | 0.11 | 0.03 | **2.29** | 0.25 | 0.30 | 0.25 | 0.25 |
|  | 33 | 0.07 | 0.06 | **2.73** | 0.25 | 0.03 | **0.74** | **6.36** |
|  | 35 | 0.08 | 0.14 | **1.57** | **0.76** | 0.23 | 0.36 | **1.24** |
|  | GMT | 0.14 | 0.11 | 1.63 | 0.80 | 0.31 | 1.02 | 0.71 |
| **B  RABITEC d.o.a.** | 3 | 0.27 | 0.47 | **4.54** | **2.02** | 0.12 | **5.94** | **0.92** |
|  | 4 | 0.25 | 0.32 | **3.27** | **0.64** | **0.56** | **1.75** | 0.24 |
|  | 23 | 0.25 | 0.25 | **1.33** | 0.36 | 0.16 | **0.57** | 0.48 |
|  | 24 | 0.25 | 0.15 | 0.09 | 0.22 | 0.07 | **1.89** | 0.09 |
|  | 37 | 0.04 | 0.05 | 0.25 | **0.99** | 0.07 | **1.87** | **0.92** |
|  | 38 | 0.03 | 0.25 | **0.79** | **0.85** | 0.23 | 0.29 | 0.25 |
|  | 39 | 0.49 | 0.14 | **1.25** | 0.37 | 0.04 | 0.25 | 0.25 |
|  | 40 | 0.04 | 0.48 | **5.50** | **0.53** | 0.17 | 0.25 | **2.55** |
|  | 41 | 0.25 | 0.25 | **2.75** | **1.97** | 0.08 | 0.19 | 0.25 |
|  | 42 | 0.03 | 0.06 | **5.76** | **1.58** | 0.37 | **2.69** | **6.83** |
|  | GMT | 0.13 | 0.19 | 1.43 | 0.74 | 0.14 | 0.85 | 0.55 |
| **C  Bayovac*R** | 1 | 0.06 | **11.66** | **39.16** | **23.29** | **7.16** | **5.01** | **2.94** |
|  | 5 | 0.25 | **2.79** | **18.74** | **19.10** | **0.88** | **2.18** | 0.25 |
|  | 6 | **0.81** | **1.00** | **7.64** | **21.34** | **2.67** | **7.32** | **2.37** |
|  | 7 | 0.02 | 0.48 | **1.73** | **0.76** | **0.57** | **1.10** | 0.42 |
|  | 9 | 0.12 | **1.43** | **25.56** | **14.27** | 0.30 | **2.71** | **1.21** |
|  | 11 | 0.25 | 0.25 | **10.87** | **8.28** | **2.79** | **5.92** | **2.55** |
|  | 14 | 0.25 | 0.10 | **12.76** | **3.11** | 0.26 | **2.19** | 0.40 |
|  | 15 | 0.16 | 0.14 | **4.24** | **4.36** | 0.39 | **0.78** | **0.78** |
|  | 18 | 0.25 | 0.34 | **4.16** | **3.18** | 0.46 | **4.51** | **2.29** |
|  | 20 | 0.25 | 0.31 | **7.79** | **3.92** | 0.37 | **1.48** | **1.86** |
|  | GMT | 0.17 | 0.62 | 9.27 | 6.54 | 0.82 | 2.63 | 1.12 |
| **D  Placebo bait** | 21 | 0.25 | 0.04 | 0.29 | 0.47 | 0.05 | **6.84** | 0.25 |
|  | 25 | 0.01 | 0.07 | 0.25 | 0.01 | 0.07 | 0.04 | 0.01 |
|  | 27 | 0.14 | 0.03 | 0.25 | 0.05 | 0.04 | 0.05 | 0.13 |
|  | 29 | 0.07 | 0.11 | 0.09 | 0.17 | 0.20 | 0.14 | 0.10 |
|  | 31 | 0.02 | 0.25 | 0.25 | **1.61** | 0.25 | 0.03 | 0.13 |
|  | 34 | 0.13 | 0.11 | 0.46 | 0.33 |  |  |  |
|  | 36 | 0.25 | 0.09 | 0.25 | 0.40 | 0.11 | 0.25 | 0.13 |
|  | GMT | 0.08 | 0.08 | 0.24 | 0.19 | 0.10 | 0.16 | 0.10 |
| **E  Control** | 43 | 0.05 | 0.11 | 0.33 | 0.03 | 0.07 | 0.25 | 0.25 |
|  | 44 | 0.15 | 0.42 | 0.20 | 0.25 | 0.12 | 0.25 | 0.25 |
|  | 45 | 0.06 | 0.15 | 0.25 | 0.25 | 0.04 | 0.40 | 0.23 |
|  | 46 | 0.22 | 0.25 | 1.87 | 0.32 | 0.06 | 0.25 | 0.12 |
|  | GMT | 0.10 | 0.20 | 0.42 | 0.15 | 0.07 | 0.28 | 0.20 |
