# Supplementary material for: Humoral Immune Response of Thai Dogs after Oral Vaccination against Rabies with the SPBN GASGAS Vaccine Strain
Source: Vaccines (Basel). 2020 Oct 1;8(4):573. doi: 10.3390/vaccines8040573 (PMC7711832; doi:10.3390/vaccines8040573)
Supplement: Supplementary file 1 [file vaccines-08-00573-s001.zip › Table S4.docx]

**Table S3.** Statistical comparison of differences in seroconversion rate between the two orally vaccinated groups (Group A vs. B – bait/d.o.a.) and the combined orally and the parenteral vaccinated group (Group A+B vs C – oral/s.c.) at different sampling points post vaccination based on both ELISA and RFFIT results.

| **dpv** | **ELISA**  **(Group A vs B -**  **bait vs d.o.a.)** | **RFFIT**  **(Group A vs B - bait vs d.o.a.)** | **ELISA**  **(Groups A+B vs C - oral vs s.c.)** | **RFFIT**  **(Groups A+B vs C - oral vs s.c.)** |
| --- | --- | --- | --- | --- |
| 7 | 5/15 vs 3/10  P=0.86, n.s. Chi²-test | 0/15 vs 0/10  n.s. Fisher’s exact test | 8/25 vs 10/10  P=0.0003 Fisher’s exact test | 0/25 vs 4/10  P=0.004 Fisher’s exact test |
| 14 | 14/15 vs 10/10  n.s. Fisher’s exact test | 10/15 vs 8/10  P=0.47, n.s. Chi²-test | 24/25 vs 10/10  n.s. Fisher’s exact test | 18/25 vs 10/10  n.s. Fisher’s exact test |
| 28 | 15/15 vs 10/10  n.s. Fisher’s exact test | 8/15 vs 7/10  P=0.41, n.s. Chi²-test | 25/25 vs 10/10  n.s. Fisher’s exact test | 15/25 vs 10/10  P=0.03 Fisher’s exact test |
| 90 | 14/15 vs 10/10  n.s. Fisher’s exact test | 4/15 vs 1/10  P=0.31, n.s. Chi²-test | 24/25 vs 10/10  n.s. Fisher’s exact test | 5/25 vs 5/10  P=0.08 Chi²-test |
| 180 | 14/15 vs 9/10  P=0.76, n.s. Chi²-test | 8/15 vs 6/10  P=0.74, n.s. Chi²-test | 23/25 vs 10/10  n.s. Fisher’s exact test | 14/25 vs 10/10  P=0.02 Fisher’s exact test |
| 365 | 13/15 vs 8/10  P=0.66, n.s. Chi²-test | 7/15 vs 4/10  P=0.74, n.s. Chi²-test | 21/25 vs 9/10  P=0.65, n.s. Chi²-test | 11/25 vs 7/10  P=0.16, n.s. Chi²-test |

1^st^ row number of seropositive animals/total number of animals per group, 2^nd^ row level of significance, 3^rd^ row statistical test used; n.s. – not significant;
